# Assessment of packed bed bioreactor systems in the production of viral vaccines

**DOI:** 10.1186/s13568-014-0025-z

**Published:** 2014-04-25

**Authors:** Ramya Rajendran, Rajendra Lingala, Siva Kumar Vuppu, Bala Obulapathi Bandi, Elaiyaraja Manickam, Sankar Rao Macherla, Stéphanie Dubois, Nicolas Havelange, Kapil Maithal

**Affiliations:** 1 Research and Development Centre, Indian Immunologicals Limited, Rakshapuram, Gachibowli, Hyderabad 500032, India; 2 ATMI LifeSciences, 10851 Louisiana Ave. S, Bloomington 55438, MN, USA; 3 ATMI LifeSciences, Rue de Ransbeek 310, Bruxelles, 1120, Belgium

**Keywords:** Vaccine production, Rabies virus, Chikungunya virus, Hepatitis-A virus, Packed bed bioreactor, iCELLis Nano bioreactor

## Abstract

Vaccination is believed to be the most effective method for the prevention of infectious diseases. Thus it is imperative to develop cost effective and scalable process for the production of vaccines so as to make them affordable for mass use. In this study, performance of a novel disposable iCELLis fixed bed bioreactor system was investigated for the production of some viral vaccines like Rabies, Hepatitis-A and Chikungunya vaccines in comparison to conventional systems like the commercially available packed bed system and roller bottle system. Vero and MRC-5 cell substrates were evaluated for growth parameters in all the three systems maintaining similar seeding density, multiplicity of infection (MOI) and media components. It was observed that Vero cells showed similar growth in all the three bioreactors whereas MRC-5 cells showed better growth in iCELLis Nano system and roller bottle system. Subsequently, the virus infection and antigen production studies also revealed that for Hepatitis-A and Chikungunya iCELLis Nano bioreactor system was better to the commercial packed bed bioreactor and roller bottle systems. Although for rabies antigen production commercially available packed bed bioreactor system was found to be better. This study shows that different bioreactor platforms may be employed for viral vaccine production and iCELLis Nano is one of such new convenient and a stable platform for production of human viral vaccines.

## Introduction

Viral vaccines are usually produced by anchorage-dependent cell lines. The use of improved modern tissue culture technology for the large-scale propagation of adherent cells is required to overcome the scalability issue. At industrial scale, these cells are either cultivated on suspended micro-carriers in bioreactors or in static mode on multiplate systems (Aunins [2000]). Microcarrier cultures require numerous complex operations from pre culture to final process, whereas, multiplate and roller bottle systems are bulky and require a lot of handling operations and susceptible to contamination (Butler [1987]). In the recent past, packed bed bioreactor systems have attracted considerable interest for the commercial production of biopharmaceuticals or vaccines. The use of fixed-bed bioreactors are known to retain and perfuse cells at high-cell densities of non-adherent and adherent cells in small reactors with low shear stress. In addition, cells in a packed-bed remain much longer viable during cultivation in an easy to operate system. The usage of packed bed bioreactors for the cultivation of anchorage dependent mammalian cells have been reported for many years (Golmakany et al. [2005]; Meuwly et al. [2004]; Kaufman et al. [2000]; Sun and Zhang [2003]). The latest generation of packed bed bioreactors used in bioprocess applications achieves very high cell densities leading to outstandingly high volumetric productivity (Meuwly et al. [2007]). This facilitates to meet the increased demand of the vaccine without having to expand the manufacturing facility. The numerous advantages of disposable bioreactors are well-known: safety of both products and operators, suppression of expensive cleaning and time consuming validation and sterilization operations. Moreover, the use of disposables increases the speed of development and the manufacturing flexibility. However, most of the currently available technologies are suitable for batch and fed-batch processes but not well adapted to viral vectors and vaccines manufacturing, as perfusion is the technology of choice for these productions (Drugmand et al. [2010]).

iCELLis Nano is a novel scalable fixed-bed disposable bioreactor system for the optimal growth of cells. It is believed to be an efficient system for high yield production of various veterinary and human viral vaccines. In fact, Drugmand et al. ([2009]) have shown that iCELLis Nano is a scale-down version of larger production units and it is a right tool to benchmark with traditional cell culture systems. Viral diseases such as Rabies, Hepatitis-A and Chikungunya are widely prevalent in most of the developing countries (Bourhy et al. [2010], Franco et al. [2012], Edelman et al. [2000]; Sreekumar et al. [2010]). Therefore, increased productivity of these viral vaccines is warranted to have low cost and affordable vaccines (Whitford and Fairbank [2011]). The latest generation of packed bed bioreactors used in bioprocess applications are expected to achieve very high cell densities leading to outstandingly high volumetric productivity. Moreover, viral replication is the real evidence to implement the process in the new platform technology. Therefore, here we have evaluated the feasibility of using the novel iCELLis Nano disposable bioreactor system for the production of some viral vaccines such as Rabies, Hepatitis-A and Chikungunya vaccine in Vero and MRC-5 cells. One of the commercially available packed bed bioreactor system and Roller bottle systems have also been included in this study for the comparative analysis.

Our results show that the titer values of Rabies were highest for the commercially available packed bed system. However, we observed two fold increases in the antigen yield and double the number of vaccines doses for Hepatitis-A and Chikungunya vaccines from iCELLis Nano in comparison to other two systems evaluated in the study. This could help to reduce the production cost and manufacturing time. This data indicates that iCELLis Nano is a convenient and stable platform for the production of Hepatitis-A and Chikungunya vaccines.

## Materials and methods

### Cell line

Vero (a continuous African Green Monkey kidney cell line) and Neuro-2a cell lines were obtained from ATCC (Manassas,USA) and grown in minimum essential medium (MEM) (Sigma, St. Louis, USA) supplemented with 10% Fetal bovine serum (FBS) (PAA Laboratories, Pasching, Austria). Human lung diploid cells, MRC-5 (Medical Research Council-5 cells) were obtained from NIBSC (Hertfordshire, UK) and maintained in MEM with Hank’s salt (H-MEM, Applichem, Tutzing, Germany) with 10% FBS.

### Virus source

The Rabies virus Pasteur strain, L 2061 (PV), used in this study is a vaccine strain obtained from Institut Pasteur, Paris, France and propagated in Vero cell line in the MEM without serum. Hepatitis-A virus (HM-175) and Chikungunya virus (CHIK 181/Clone 25) were received from Centre for Disease Control, Atlanta and United States Army Medical Research Institute of Infectious Diseases, Frederick, MD respectively. These viruses were propagated in MRC-5 cells Virus maintenance medium (VMM) which consisted of H-MEM medium with 3% FBS, (Moregate, Australia).

### Bioreactors

iCELLis Nano bioreactor (ATMI Life Sciences, Bruxelles, Belgium) is a disposable bioreactor pre-filled with proprietary macrocarriers entrapped in a 200mL fixed-bed. The commercially available packed bed bioreactor system (undisclosed) was also crammed with carriers to get the same surface area as of iCELLis Nano system. Whereas, the roller bottles of 850 cm^2^ surface area (Corning, Lowell, MA) equivalent to the surface area of the carriers in packed bed systems were used in the study to normalize the surface area for all the three bioreactor systems. Additionally, similar initial seeding density, multiplicity of infection (MOI) and media components used in all the three systems in the production process of Rabies, Hepatitis-A and Chikungunya vaccines respectively were used.

### Sampling and metabolite analysis

Carriers were sampled out (in triplicate) at the same time points from iCELLis Nano and commercially available packed bed bioreactor systems and the cell counts were measured using crystal violet staining. The cell counts from roller bottles were also measured at same time intervals using trypan blue dye exclusion method. Residual glucose and accumulated lactate concentrations in the spent media were also monitored using an YSI 2700 select analyzer (YSI incorporated Life sciences, Ohio, USA).

### Cell seed preparation

The cells (Vero and MRC-5) were first grown in monolayer using the respective media at 37° C and were detached after reaching confluence by standard trypsinization protocol and inoculated into bioreactor to start the bioreactor culture.

### Growth kinetics of Vero and MRC-5 cells

Initial experiments were done to see the growth kinetics of Vero cells in packed bed systems in comparison with roller bottles. The cell counts were compared on day five, after reaching 100% of confluence and cell densities were compared for all the three systems.

Similarly, the growth kinetics of MRC-5 cells was compared in packed bed systems and roller bottles by measuring the cell counts on day six, after they reached 100% confluence. The cells per cm^2^ surface area of the carriers was calculated and compared with cell count/ cm^2^ surface area of the roller bottle system. The initial inoculation density was maintained same for all the three systems in both the studies

### Process for the production of rabies vaccine

In the infection study, Vero cells were infected with Rabies virus and grown onto the carriers of iCELLis Nano and commercial packed bed bioreactor systems by perfusion mode. Perfusion was set up one day after the start of culture. Glucose and lactate levels were analyzed using the YSI 2700 select analyzer. Perfusion flow rate for the packed bed systems was adjusted in such a way so as to maintain glucose level above ~1 g/L and the lactate level below ~1.5 g/L in the spent media. Vero cells grown on roller bottle system were also infected with Rabies virus using the same MOI by batch mode. Multiple viral harvests were taken from the infected Vero cells until day sixteen post infection (PI) as per the standard protocol required for the production of Rabies vaccines. Virus production was measured by titration in Neuro-2a cell line and expressed as tissue culture infectivity dose_50_/ml (TCID_50/_ml). Finally, the cumulative TCID_50_ titer of the collected viral harvests and the total media consumption were compared for all the three systems used in this study.

### Process for the production of Hepatitis-A vaccine

The infection study was initiated after comparing the growth kinetics of MRC-5 cells in all the three systems used in this study. Briefly, MRC-5 cells were grown onto the carriers of iCELLis Nano and commercial packed bed bioreactor systems in perfusion mode. Spent media was analyzed periodically for glucose and lactate levels. Perfusion was started 24 hrs after the start of culture and the perfusion rate was modulated in such a way so as to maintain the glucose level above ~0.1 g/L and the lactate level below ~1.5 g/L in the spent media. The cells were infected with Hepatitis-A virus on day five, after reaching 80 to 90% of confluence on the carrier. On day five, the cell growth medium (H-MEM with 10% FBS) was replaced with VMM. Similarly, MRC-5 cells grown on roller culture bottles were also infected with Hepatitis-A virus on day five at the same MOI and maintained by batch mode. The spent media in roller bottles were replenished with fresh VMM at an interval of seven days. The infected cultures were maintained and the virus was harvested from the infected cells on day 21 post infection (PI). The antigen yield was quantified using the HAV antigen ELISA kit (Mediagnost, Reutlingen, Germany).

### Process for the production of Chikungunya vaccine

Human diploid cell line, MRC-5 was grown onto the carriers of iCELLis Nano and commercial packed bed bioreactor systems in perfusion mode. Once the cells reached a confluence of 80-90% by day five, they were infected with Chikungunya virus. Perfusion was started 24 hrs after the batch initiation and the perfusion flow rate for the packed bed system was adjusted according to cell density and was gradually increased during the culture period to maintain the glucose level above ~0.1 g/L and the lactate level below ~1.5 g/L in the spent media. Cells grown on roller bottles were also infected similarly on day five with Chikungunya virus using the same MOI by batch mode. After infection, three harvests were taken from all the three systems on different days by replenishing with fresh VMM (as per the standard optimized internal production protocol of Chikungunya vaccine). The harvested virus was titrated on Vero cells and the titers of harvests were calculated as plaque forming units/ml (pfu/ml).

## Result

### Cell growth kinetics

The growth kinetics of Vero and MRC-5 cells were studied in iCELLis Nano, a commercial packed bed fermenter and roller bottle systems to evaluate the suitability of the bioreactor systems for large scale propagation of these cell lines. Vero cells grown on the carriers of iCELLis Nano and the packed bed systems reached the highest count of ~ 4 x10^6^ cells/ml of bed on day five, which was a ~10 fold increase from the number of cells inoculated. Similarly, cells grown on the roller bottles (with equivalent surface area) also showed ~10 fold increase in the count by fifth day. These results clearly show similar growth kinetics of Vero cells in all the three systems. Whereas, for MRC-5 cells, the cell density achieved was better in the iCELLis Nano and roller bottle systems as compared to the commercially available packed bed system (Table 1).

**Table 1:** Comparison of growth kinetics of MRC-5 cells in rollers, iCELLis Nano and packed bed system

	**Rollers**	**iCELLis Nano**	**Packed bed system**
Number of cells/cm^2^ of surface area	0.31 x 10^5^	0.26 x 10^5^	0.1 x10^5^

### Production of rabies vaccine

Comparative analysis of the virus titers obtained in the three bioreactor systems revealed that the commercial packed bed system had a better cumulative titer of ~ 4.3 x 10^10^ TCID_50_, which was almost one log higher than the iCELLis Nano and roller bottle systems, which had titers of ~1.8 x 10^9^ TCID_50_ and ~ 1.2 x 10^9^ TCID_50_ respectively (Table 2). Further it was found that the commercial packed bed system utilized 2.5 to 3.5 times less media than the other two systems.

**Table 2:** **Comparison of media consumption and cumulative TCID**_**50**_**titer of the three different systems evaluated in this study for the process of Rabies vaccine production**

**System name**	**Total media used (L)**	**Cumulative titer (TCID**_**50**_**)**
Roller culture system	22	~1.2X10^9^
iCELLis Nano	33	~1.8X10^9^
Packed bed system	9	~ 4.3X10^10^

### Production of Hepatitis-A vaccine

**Figure 1:**
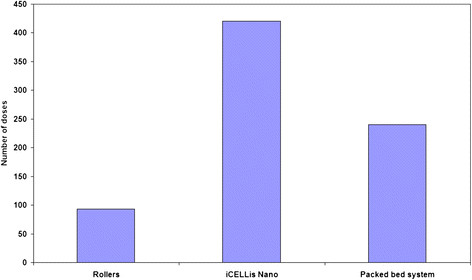
Comparing the number of vaccine doses obtained from Rollers, iCELLis Nano and packed bed system for the production of Hepatitis-A vaccine.

**Figure 2:**
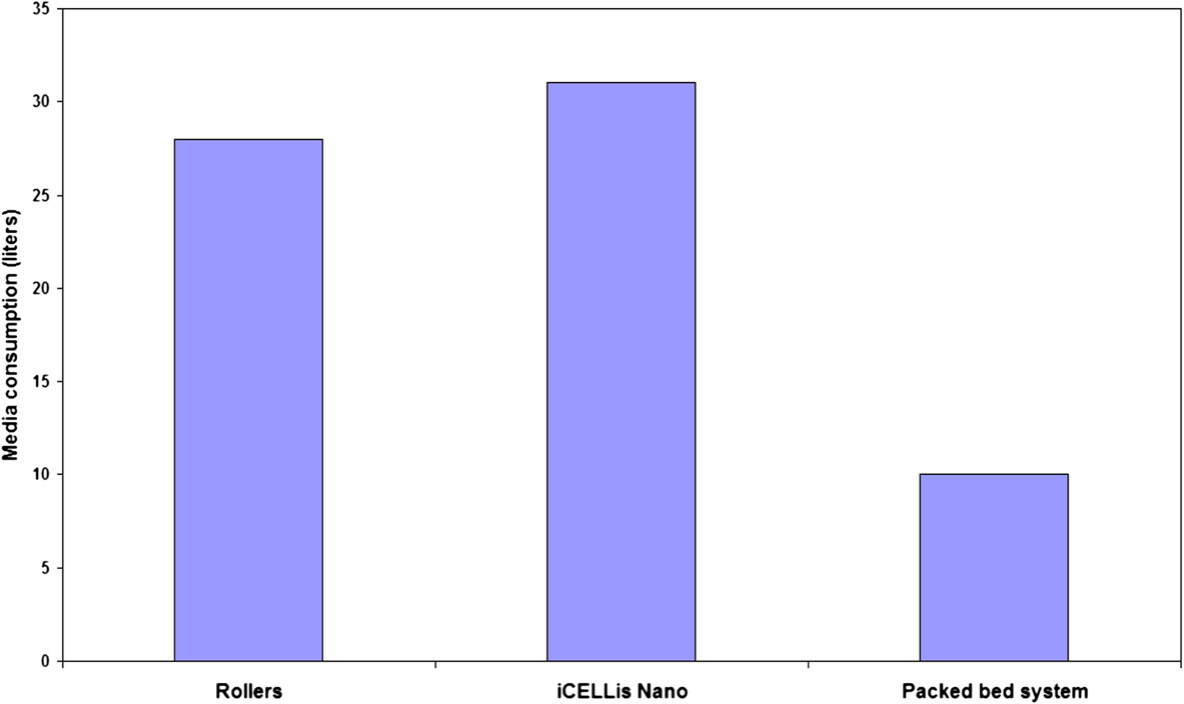
Comparison of media consumption for the process of Hepatitis-A vaccine production using rollers, iCELLis Nano and packed bed system.

The virus harvest was collected on day 21 PI from all the three systems and the antigen yield in the collected harvest was quantified. It was observed that the production of Hepatitis-A antigen was almost two-fold higher in iCELLis Nano bioreactor system as compared to the other two systems indicative of higher number of doses in iCELLis Nano system (Figure 1). But consistent with the Rabies data the commercial packed bed bioreactor system utilized 2-3 folds less media in comparison to other two systems (Figure 2).

### Production of Chikungunya vaccine

**Figure 3:**
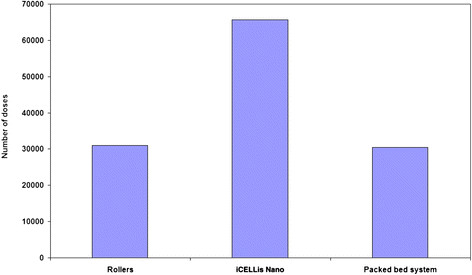
Comparing the number of vaccine doses obtained from Rollers, iCELLis Nano and packed bed system for Chikungunya vaccine.

**Figure 4:**
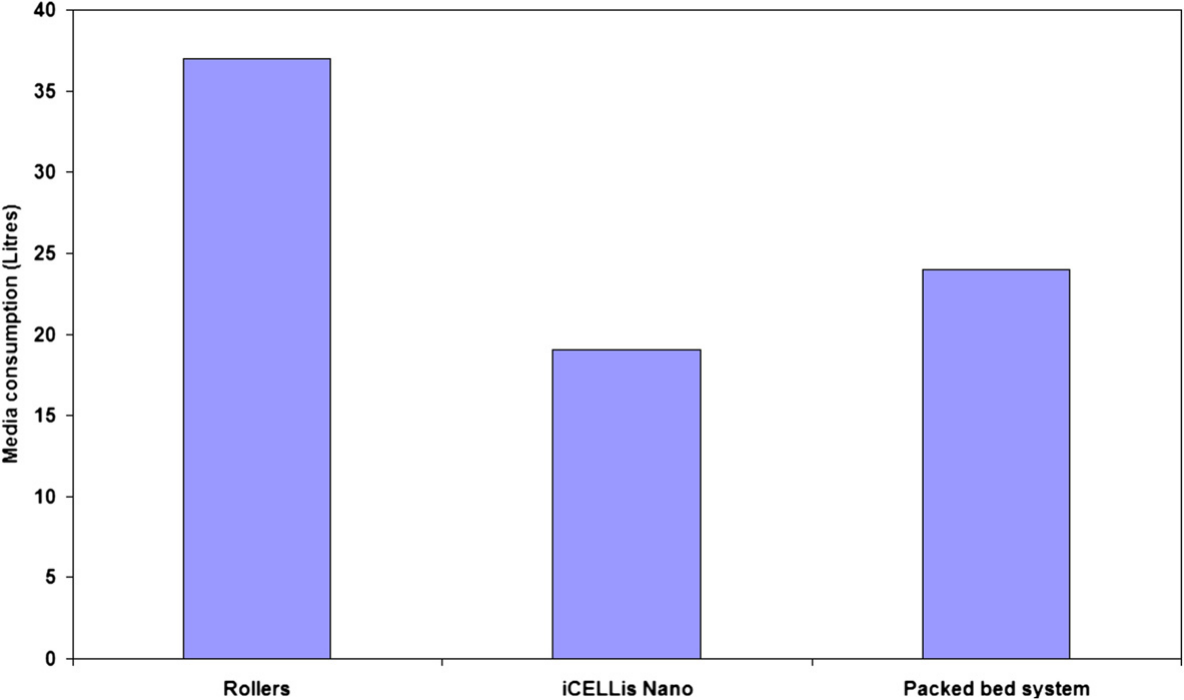
Comparison of media consumption for the process of making Chikungunya vaccine in rollers, iCELLis Nano and packed bed system.

The post infection harvests were collected and titrated using Vero cell line. The harvest volumes were maintained constant for all the three systems for comparison. Titration results showed that overall productivity was superior in iCELLis Nano as compared to other two systems, whereas there was a two log reduction in the virus titers in the third harvest of rollers in comparison to iCELLis Nano and packed bed system (Table 3). Indeed, this translated into two time higher vaccine doses obtained from the iCELLis Nano bioreactor system as compared to other two systems (Figure 3). Additionally, the iCELLis Nano bioreactor system consumed least media in comparison to other two systems evaluated in this study for Chikungunya vaccine development (Figure 4).

**Table 3:** Chikungunya virus Titers for each harvest from iCELLis Nano, commercial packed bed system and roller culture bottles

**Harvest Number**	**iCELLis Nano**	**Commercial packed bed system**	**Roller culture bottles**
	**(Titer pfu/ml)**	**(Titer pfu/ml)**	**(Titer pfu/ml)**
I	3.2 x10^7^	5.6 x 10^6^	2 x 10^6^
II	4x10^7^	2.4 x 10^6^	0.1 x 10^6^
III	2x10^6^	1.8 x 10^6^	2.8 x 10^4^

## Discussion

Production of cost effective vaccines is of prime importance to eradicate majority of viral diseases in developing countries. Cell lines used for production are particularly difficult to grow and various bioreactors offer options for optimizing growth conditions to maintain cell viability and increase in productivity (Whitford and Fairbank [2011]). Current, packed bed systems could help to yield high cell density with immobilization of cells into a 3D matrix. Therefore, use of packed bed bioreactors in bioprocess applications could help in achieving very high cell densities leading to high volumetric productivity, which in turn will reduce the manufacturing cost of the product. In addition, packed bed systems have numerous advantages when compared to traditional cell culture systems like reduction of footprint for equivalent production capacity, decrease of operational costs by providing large scale platform, flexibility to increase production capacity, automation and integration into a fully closed manufacturing process. The use of bioreactor system for the production of vaccines also allows measurements; control and supervision of process parameters like dissolved oxygen, pH and temperature in real time unlike stationary cultures (Sun et al. [2013]). This is particularly valuable in present regulatory environment where authorities request more details of the biotechnological processes utilized in product development.

The disposable bioreactor systems provide similar growth and productivity as stainless steel bioreactors. In addition, the use of disposable systems have faster turnaround time between batches due to reduction of cleaning and validation processes, which are costly and time consuming steps in the manufacturing process. Thus use of disposable systems in production of vaccines could help to meet the unpredictable demand without having upfront capital investment in the manufacturing unit. Drugmand et al. ([2009,2010]) have earlier demonstrated that iCELLis is an efficient and validated novel scalable fixed-bed disposable bioreactor system for the high volumetric productivity of human and animal viral vaccines. In order to evaluate the feasibility of using this system in our production process, we have assessed the iCELLis Nano, a scale-down version of larger production unit, in the production of Rabies, Hepatitis-A and Chikungunya viral vaccines in comparison with the other commercially available packed bed system and the conventional roller bottle system.

Availability of new low density matrices in recent times, allow up scaling of adherent cells in bioreactors for vaccine production (Whitford and Fairbank [2011]). The results of Vero cell growth kinetic study showed a ~10 fold increase of total cell mass in iCELLis Nano and packed bed bioreactor systems and the increase in cell mass obtained was comparable with roller bottle system. This data indicates that the packing material used in iCELLis Nano could support the cell growth satisfactorily in comparison to similar like other systems. Though Vero cell growth kinetics is similar for all the three platforms evaluated, the cumulative titer obtained for Rabies virus using the commercial packed bed system had the highest TCID_50_ titer. Comparatively superior growth performance of MRC-5 cells on the carriers of iCELLis Nano could credit for the two times higher number of vaccine doses obtained for Hepatitis-A and Chikungunya vaccines. The increase in vaccine doses has the advantage of cost reduction and also decreases the manufacturing time to meet the demand, which is critical in pandemic outbreaks. This data clearly confirms that iCELLis Nano is a suitable fixed bed system, which can support the growth of MRC-5 cells and provides favorable conditions for the growth of Hepatitis -A and Chikungunya viruses. This also demonstrates the suitability of iCELLis Nano for continuous perfusion operations and does not require manual culture media exchange. Therefore, it reduces the chances of contamination and maximizes the antigen yield by supplying optimal media requirements. In addition, the harvesting process of Chikungunya and Hepatitis-A may require extraction of virus from the infected cells (Heinricy et al. [1987]; Peters and Dalrymple [1990]), whereas, the release of Rabies virus is by budding process and does not require cell lysis (Mebatsion et al. [1999]). Thus the higher antigen yields observed for Hepatitis-A and Chikungunya vaccines using iCELLis Nano system could possibly be attributed to this phenomenon indicating that iCELLis Nano may be an ideal system for the production of viral vaccines in which cell lysis is one of the important process step. In summary, this study clearly demonstrates that the new iCELLis Nano system may provide the ease of scalability thus reducing optimization time on scaling up the process to manufacturing scales and improving the productivity of currently manufactured and new viral vaccines. Although assessment with other viruses may be required to validate the use of this system as a common platform for viral vaccine production.

## Competing interests

The authors declare that they have no competing interests. Stéphanie Dubois is an ATMI LifeSciences paid employee and Nicolas Havelange is a paid consultant for ATMI LifeSciences.

## Authors’ contributions

KM reviewed the data and the manuscript. RR designed and executed the study for Rabies, performed virus quantification assays for Rabies, Hepatitis-A and Chikungunya and wrote the manuscript. RL designed and executed the study for Hepatitis-A and Chikungunya . SKV and BOB performed the Bioprocess for Hepatitis-A and Chikungunya. ME performed the Bioprocess for Rabies virus. SRM performed the chromatography. SD and NH designed the study in iCELLis Nano bioreactor. All authors read and approved the final manuscript.
